# Superb microvascular ultrasound is a promising non-invasive diagnostic tool to assess a ventriculoperitoneal shunt system function: a feasibility study

**DOI:** 10.1007/s10143-024-02665-w

**Published:** 2024-09-02

**Authors:** Konstantin Brawanski, Ondra Petr, Christian Preuss Hernandez, Nikolaus Kögl, Claudius Thomé, Elke R. Gizewski, Hannes Gruber, Michael Verius, Leonhard Gruber, Daniel Putzer

**Affiliations:** 1 Department of Neurosurgery, Medical University Innsbruck, Anichstrasse 35, Innsbruck, 6020 Austria; 2 Department of Radiology, Medical University Innsbruck, Anichstrasse 35, Innsbruck, 6020 Austria

**Keywords:** Superb microvascular ultrasound, Sonography, Ventriculoperitoneal shunt, Optical nerve sheath diameter, Papilla diameters, Flow

## Abstract

The objective of this pilot study was to assess the reliability of superb microvascular ultrasound (SMI) for the measurement of the cerebrospinal fluid (CSF) flow within VPS systems as an indirect sign for shunt dysfunction. Asymptomatic hydrocephalus patients, with a VPS system implanted between 2017 and 2021, were prospectively enrolled in the study. Using SMI, the CSF flow within the proximal and distal catheters were analysed. Before and after pumping the shunt reservoir, intraabdominal free fluid, optical nerve sheath diameter (ONSD), and papilla diameter (PD) were evaluated and correlated with the amount of valve activation. Nineteen patients were included. A flow was detectable in 100% (*N* = 19) patients in the proximal and in 89.5% (*N* = 17) in the distal catheter. The distal catheter tip was detectable in 27.7% (*N* = 5) patients. Free intraabdominal fluid was initially detected in 21.4% (*N* = 4) patients and in 57.9% (*N* = 11) at the end of the examination (*P* = 0.049). ONSD was significantly lower after pump activation (4.4 ± 0.9 mm versus 4.1 ± 0.8 mm, *P* = 0.049). Both peak velocity and flow volume per second were higher in proximal compared to distal catheters (32.2 ± 45.2 versus 5.6 ± 3.7 cm/sec, *P* = 0.015; 16.6 ± 9.5 ml/sec versus 5.1 ± 4.0 ml/sec, *P* = 0.001, respectively). No correlation was found between the number of pump activations and the changes in ONSD (*P* = 0.975) or PD (*P* = 0.820). SMI appears to be a very promising non-invasive diagnostic tool to assess CSF flow within the VPS systems and therefore affirm their function. Furthermore, appearance of free intraperitoneal fluid followed by repeated compression of a shunt reservoir indicates an intact functioning shunt system.

## Introduction

Ventriculoperitoneal shunt (VPS) dysfunctions represent a true emergency, conceivably terminating lethally [1–4]. VPS have a high failure rate up to 40% within the first year after initial implantation and up to 98% within the next 10 years [5–7]. The most common complications are infections and mechanical dysfunction [3, 8, 9].Symptoms indicating this serious condition oftentimes vary extensively from headache, nausea and emesis to coma and death. Correspondingly, patients may deteriorate rapidly with far-reaching aftermaths [10–12]. Importantly, 10% of all fatalities in children with implanted VP-shunts are due to a shunt dysfunction [13]. In order to rule out VPS malfunction, multimodal emergency cranial imaging is necessary to evaluate e.g. ventricular size [14]. Plain radiographic shunt series exclude disconnections or malposition and assess the valve setting in case of programmable systems [15]. Elevated intraventricular pressure without ventriculomegaly can be found in long-term VPS patients with long-term inserted VPS leading to a reduced ventricular compliance with no or minimal change of the width of the ventricular system in cranial imaging despite elevated intracranial pressure [16]. This possibility in a rapidly deteriorating symptomatic patient along with the absence of ventriculomegaly calls for explorative surgery and VPS revision. This may cause frustration of patients as well as physicians [1, 2] as revision surgery is associated with an high rate of subsequent VPS dysfunctions and infections [2, 17, 18].Hence, a strong call for reliable, predominantly non-invasive diagnostic tools has ascended recently. Being on the front burner, ultrasound (US) especially the contemporaneous advanced devices and techniques such as measuring of the optic nerve sheath diameter (ONSD), enables to steer coincident trend in diagnostics and treatment. Notably, none of these modern non-invasive methods available is embodied in the current standards of care yet. Using these in a preoperative diagnostic approach, especially as a bundle of non-invasive diagnostic procedures available may substantially expedite already very demanding time-consuming task and thus ameliorate patient outcomes in a real-world setting.Utilizing one of the contemporary advanced US techniques available, the aim of this feasibility study was to measure the cerebrospinal fluid (CSF) flow within the VPS system using US with a special focus on low velocity flow after compressing the reservoir chamber in asymptomatic patients regardless of the indication of VPS implantation. This technique may spare future surgical explorations when comparing the current value to the patients’ baseline.

## Materials and methods

A single-center feasibility study was initiated to evaluate the potential of an additional ultrasound examination in VPS patients. This study was approved by the local ethics committee of Medical University Innsbruck, Austria (AN EK Nr:1300/2020) and was initiated in 2021.Detailed information including demographics, medical history, radiographic data and clinical presentation were collected. Medical history included the etiology of hydrocephalus, prior surgeries and the type of shunt system implanted. During the clinical check-up disease-related data (i.e., headache, nausea, dizziness, meningism, vomiting, stomachache, reduction of vigilance, orthostatic hypotension, scarring, infect signs) were extracted and a detailed neurologic examination was performed.

### Radiographic data

A Canon Aplio i800 device (Tokyo, Japan) was used, performing SMI ultrasound with an 11 MHz linear array transducer. A superb microvascular imaging (SMI) standard imaging protocol was created directly on the Aplio i800 device, in accordance with Canon application specialists, named “VP shunt”. Of note, SMI is a novel US imaging technique enabling the visualization of low velocity blood flow in small vessels. The essential novel technical development of this new US procedure is based on the fact that noise generated from motion artifacts can be completely suppressed. The images include a monochrome color map of blood flow, which is registered onto the B-mode standard US image. The detection of flow within the catheter system was demonstrated by Putzer et al. in a separate experiment [21].Additionally, in the study we used ultrasonographic measurement of optic nerve sheath diameter (ONSD) for screening of suspected increased intracranial pressure (ICP) as the swelling of the optic nerve inside the eye, referred to as papilledema, is a known indicative parameter of increased ICP.The optic nerve was measured with the 11 MHz linear array transducer of the Canon Aplio i900 US device (Tokyo, Japan) in B-mode. The patients were in a supine position. Ultrasound gel was applicated on the closed eyelid and the probe was placed gently on top, avoiding increased manual pressure. Measurements of the ONSD were performed by an anterior transbulbar approach at a depth of 3 mm to the retinal surface on both sides.This technique enables fast and non-invasive assessment of a surrogate parameter for ICP.The ONSD was measured using the recently published suggested CLOSED Protocol for sonographic assessment of the optic nerve sheath diameter [22] and the papillary height was measured using the diagnostic protocol of Ebraheim et al. [23].

### Clinical and radiological examinations of the ventriculoperitoneal shunt system

All patients received a clinical examination with further imaging only in case of new clinical manifestations. This included disease-related data, e.g., headache, nausea, dizziness, meningism, vomiting, stomach ache, reduction of vigilance, orthostatic hypotension, scarring, and infect signs.The function of the reservoir was checked by pushing the valve. In a next step the ventricular catheter was closed by pushing on the ventricular catheter (directly before the valve) and activating the reservoir. In pristine condition the reservoir is squeezed out with locking the ventricular catheter and the refilling of the pump chamber is evaluated after release of the ventricular catheter. The filling phase of the reservoir after release was assessed and characterized by followed: the refilling of the reservoir was prompt, delayed (> 5 s), or there was no refilling. The presence of a flow pattern upon SMI ultrasound was used to verify a normally functioning shunt system. In case of absence of a flow wave pattern, the ultrasound transducer was placed on the proximal and on the distal catheter for the purpose of differentiating proximal from distal shunt obstruction.

### Statistical analysis

All data were collected and stored in Microsoft Excel (Microsoft Corp.; Redmond, USA). Statistical analysis was performed in GraphPad Prism 9.1.1 (GraphPad Software Inc.; La Jolla, USA). Continuous data are presented as box-plots including median and lower/upper quartiles, whiskers denote 5% and 95% percentiles; outliers are presented as circles. 95% confidence intervals (CI) are given where appropriate. P-values < 0.05 were considered significant, those < 0.1 a trend.Contingency tables were analyzed via χ [2] tests. ONSD and papilla diameters between left and right eye were compared via a linear correlation analysis, results are given as slope (95% confidence intervals in brackets), R [2] and p-values. Further linear correlation analyses were carried out between optical nerve sheath and papilla diameters before and after pump activation as described above. Continuous data was compared via a paired t-test, when comparing contralateral sides.

## Results

### Patients

A total of 19 patients were prospectively enrolled in the study. All patients had received a VPS between October 2017 and March 2021. The average age of the patients was 57.3 ± 13.5 years (range 29 to 75 years), and 15 patients were females (83.3%). All patients (*N* = 19) harbored a VPS shunt with a programmable valve together with a pumpable reservoir. Used valves, clinical symptoms and causes of hydrocephalus are listed in Table 1. On average, patients had 79.3 ± 17.3 kg body weight, 166.1 ± 7.9 cm body height and a BMI of 28.7 ± 6.2 kg/m^2^. The mean time from shunt implantation to US investigation of the VPS systems was 28.5 ± 21.4 months with 3 (15.8%) patients after additional catheter revision 17.3 ± 20.1 months prior to the current examination. Three (15.8%) patients received further imaging after onset of new deteriorating symptoms. Two (10.5%) patients received a cranial computed tomography (CT) and X-ray of the shunt valve (for confirmation of the initial setting), and the ventricular as well as the peritoneal catheters to exclude any disconnections. One (5.3%) patient received a cranial CT scan only. All patients needed no further intervention, and the US data was not used for decision-making.

**Table 1:** Clinical condition and implanted systems

**Implanted systems** (*n* = 19)	**Numbers (%)**
Medos-Hakim (programable valve)	11 (57.9%)
Certas (programable valve)	6 (31.6%)
Certas plus (programable valve)	2 (10.5%)
**Clinical symptoms** (*n* = 19)	**Numbers (%)**
clinical asymptomatic	14 (73.7%)
Headache	1 (5.3%)
Headache, nausea, dizziness, stomach pains	1 (5.3%)
Headache, dizziness	1 (5.3%)
Headache, nausea, dizziness	1 (5.3%)
hakim trias	1 (5.3%)
**Indication for hydrocephalus** (*n* = 19)	**Numbers (%)**
Subarachnoid haemorrhage	12 (63.2%)
Normal pressure hydrocephalus	3 (15.8%)
Aqueduct stenosis	1 (5.3%)
Pseudotumor cerebri	1 (5.3%)
CSF accumulation after cranial surgery	1 (5.3%)
Neurosarcoidosis	1 (5.3%)

### General examination parameters, clinical conditions & implanted systems of patients

The average valve-pressure setting was 124.5 ± 17.8 mmH_2_O (90–140 mmH_2_O). The valve mechanism was activated 39.1 ± 16.4 times on average (range 14 to 80 times). Fourteen (73.7%) patients were asymptomatic, whereas five (26.3%) of the patients presented with temporary clinical symptoms. The clinical symptoms and implanted valve types are listed in Table 1.

### Mechanical behavior and inspection of the valve

All 19 (100%) implanted valves were easy to compress and showed a repeatable prompt release after pumping the reservoir of the valve. With manual occlusion of the ventricular catheter (before the valve) and pumping the reservoir, 14 (73.7%) reservoirs stayed dented and filled up immediately after release of the ventricular catheter. In five (26.3%) patients the ventricular catheter could not be compressed.

### CSF flow within the ventriculoperitoneal shunt system

**Fig. 1:**
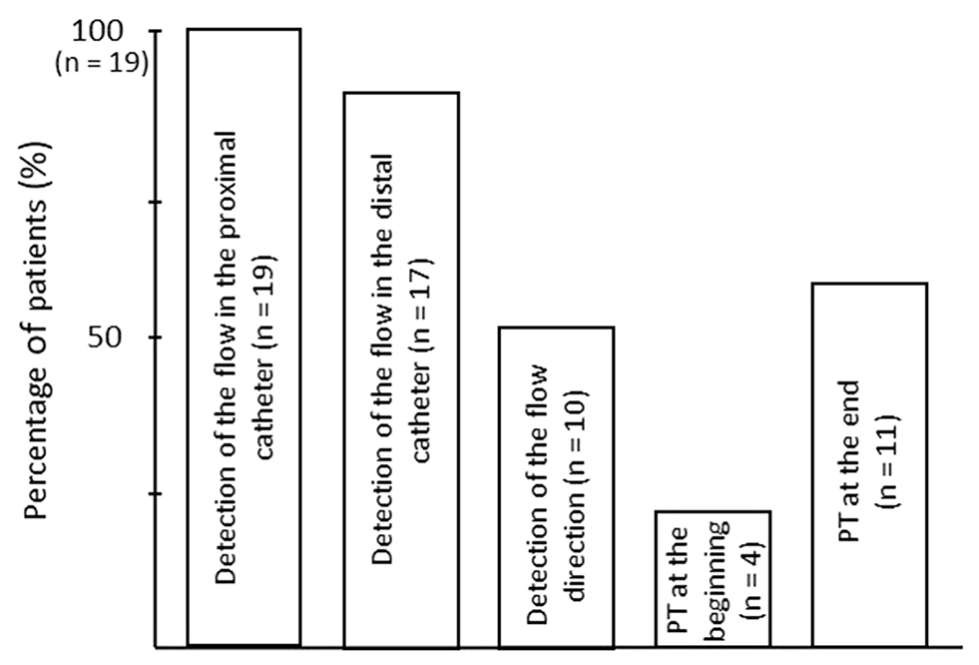
Overview of detection of fluid and flow direction (PT = peritoneal fluid)

A flow was detected in 19 (100%) patients in the proximal catheter in and in 17 (89.5%) patients in the distal catheter direct above the clavicula of the VPS (Fig. 1).

CFS flow direction was verified in 10 (52.6%) patients, in all these cases showing a cranial to peritoneal flow direction. There was a significant difference in peak velocity between the proximal and distal shunt Sect. (32.2 ± 45.2 vs. 5.6 ± 3.7 cm/sec, *P* = 0.015) as well as the flow volume per second (16.6 ± 9.5 mL/sec vs. 5.1 ± 4.0 ml/sec, *P* = 0.001) (Fig. 3).

**Fig. 2:**
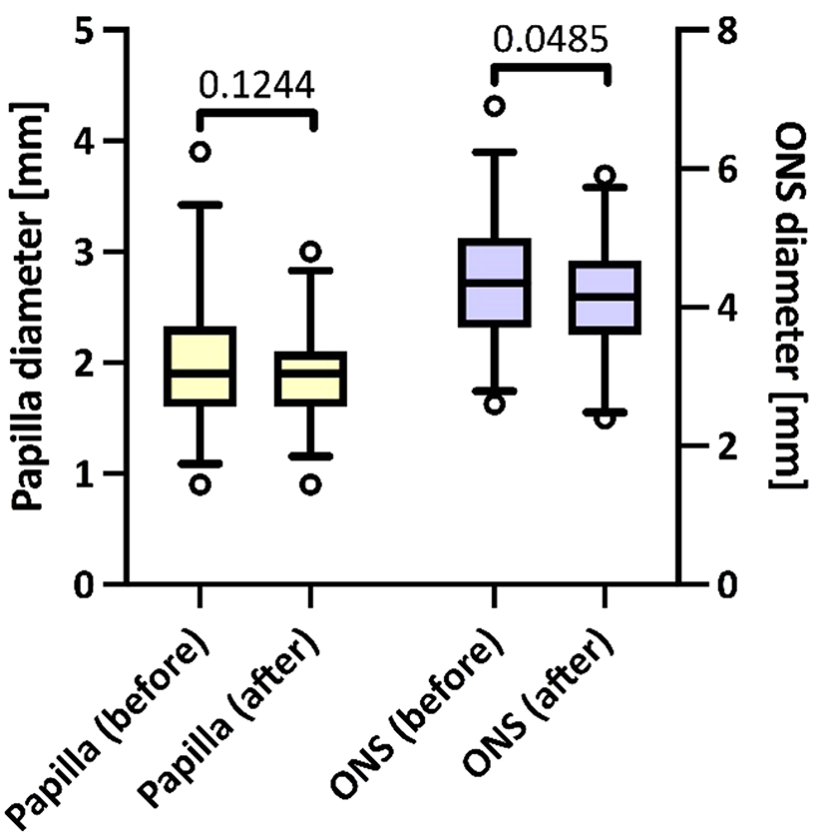
Change of ONSD and PD before and after valve activation

**Fig. 3:**
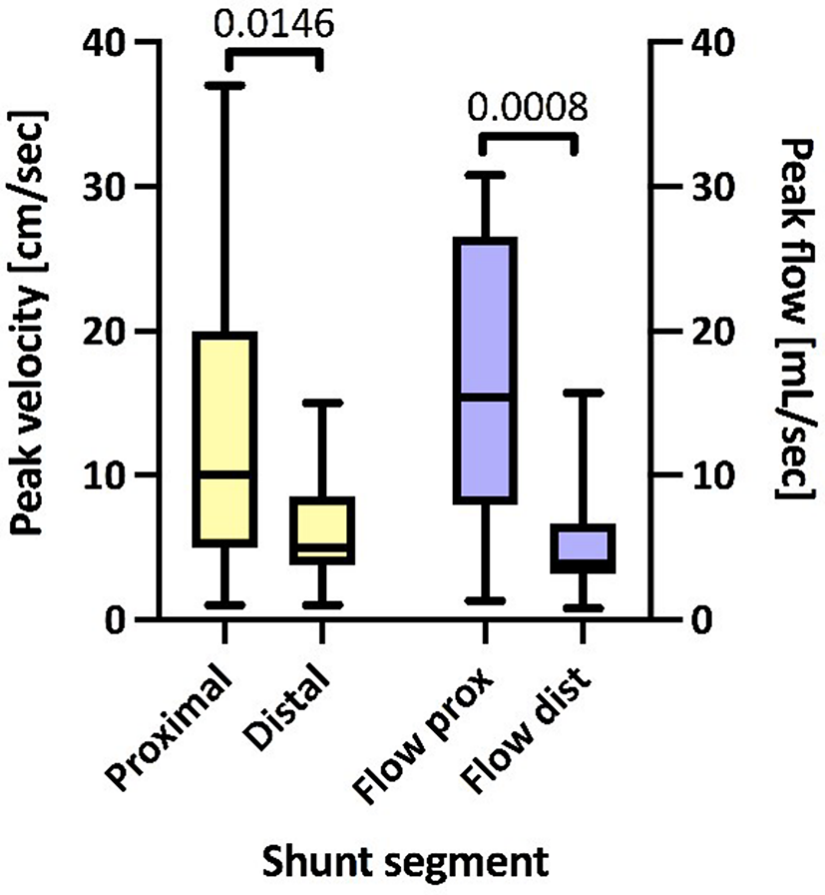
Comparison of peak velocity in the proximal and distal shunt segment during pump activation

### Visualization of the distal catheter tip and free intraabdominal fluid

The peritoneal catheter tip was visualized by ultrasound in 5 (26.3%) patients. Free intraabdominal fluid was detected in 4 (21.1%) patients in the beginning of examination and in 11 (57.9%) patients after valve activation, respectively (*P* = 0.049) (Fig. 1).

### Optical nerve sheath diameter

ONSD were significantly lower after reservoir activation (4.4 ± 0.9 mm vs. 4.1 ± 0.8 mm, *P* = 0.049) (Fig. 2). There was a strong correlation between the left and right ONSD before activation (slope 0.91 [95% CI 0.62 to 1.19], R [2] 0.72, *P* < 0.0001), which was significantly lower after pump activation (slope 0.84 [95% CI 0.10 to 1.57], R [2] 0.27, *P* = 0.028). The correlation between the bilateral ONSD before and after pump activation was moderate (slope 0.63 [95% CI 0.42 to 0.84], R [2] 0.53, *P* < 0.0001).

### Papilla diameter

PD did not change after pumping the reservoir (2.0 ± 0.6 mm vs. 1.9 ± 0.5 mm, *P* = 0.124) (Fig. 2). Left- and right-sided PD highly correlated before (slope 0.87 [95% CI 0.51 to 1.24], R [2] 0.60, *P* = 0.0001) and after (slope 0.87 [95% CI 0.60 to 1.14], R [2] 0.75, *P* < 0.0001) valve activation, respectively. The correlation between the bilateral PD before and after pump activation was moderate (slope 0.54 [95% CI 0.37 to 0.71], R [2] 0.55, *P* < 0.0001).

### Correlation between numbers of valve activation and ONSD / PD

The valve mechanism was activated 39.1 ± 16.4 times (range 14–80 times). No correlation was found between the number of activations and the changes in ONSD (*P* = 0.975) or papilla diameters (*P* = 0.820), respectively.

## Discussion

Our prospective monocentric pilot study of consecutive patients with previously implanted programmable VPS due to symptomatic hydrocephalus uses a novel technology of superb microvascular ultrasound (SMI) to closely appraise the integrity and functionality of an inserted shunt system in a completely non-invasive manner. Our data demonstrate that this technique is capable of detecting CSF flow in the overwhelming majority of shunt patients, in our study in all patients in the proximal part, and in approximately 90% of patients in the distal part of the implanted shunt system. Moreover, flow volume as well as peak velocity could be measured and determined in detail, but depends on the angle of the probe, angle correction, settings on pulse repetition frequency [24]. Surprisingly, we found a higher peak velocity in the proximal parts of the shunt system compared to the distal ones. Free intraabdominal fluid was initially detected in approximately one fifth of patients, after active pumping of the shunt reservoir ascending to approximately two thirds of patients. This maneuver also significantly reduced the optic nerve sheath diameter. Our data strongly suggest that using this ultrasound technique, dysfunction of an implanted shunt system can be adequately detected and appraised in a non-invasive fashion.Ability to detect and measure the flow along with its quantification even in case of infinitesimal flow rates within an implanted VPS has been enabled owing to novel and constantly developing advancements in imaging techniques such as SMI ultrasound with special ultrasound probes [21]. Major merits of sonographic examinations are their cheapness and availability, no radiation, and the possibility to execute these bed-side [25]. Yet, the quality and value of the examination remains highly examiner reliant [26]. As for now, ultrasound has been routinely used to identify free intraabdominal fluid as an indirect sign of an intact shunt system [27]. Notably, the absence of free fluid within the abdomen represents certainly no proof of malfunction [27, 28]. In addition, the evidence of free intraabdominal fluid may also be physiological in women, children and young men [29]. In addition, there are several studies verifying the intact VPS function using ultrasound (US) by ultrasonically excited bubbles or by a characterization of valve interface oscillation [19, 20]. Putzer et al. created one of these models to detect very low flows and flow directions within catheters using ultrasound (US) [21]. In our study, we visualized the peritoneal catheter tip in one fourth and free intraabdominal fluid in one fifth of patients. Importantly, after active pumping of the shunt reservoir, this maneuver can indirectly verify the functionality of the shunt system. Subsequently, we found the intraabdominal fluid in approximately two thirds of patients, indicating a completely intact shunt system.It is worthy of notice that sonography has also been used to measure the diameter of the optic nerve sheath (ONSD), which simultaneously changes with rising, or decreasing ICP [30–32]. The measurement of ONSD appears to be more meaningful in case of a rapid elevation of ICP in comparison to the measurements of a congested papilla, which may be missing due to a delayed occurrence [33–36]. We were able to show a significant decrease of the ONSD after pump activation at the end of our examinations, whereas the measured papilla diameters did not change. Interestingly, there was no correlation between the number of pump activations and the changes in both ONSD and papilla diameters. Of note, the time course for development of papilledema varies widely, mostly depending on the slope of the ICP rise. Compensatory mechanisms may preclude the development of papilledema in chronic cases [37]. It is worthy of notice that the measurement of ONSD has emerged as a valuable non-invasive tool also in normal pressure hydrocephalus (NPH). In NPH, despite the typically normal baseline cerebrospinal fluid (CSF) pressures, fluctuations in ICP can occur, particularly during postural changes or transient CSF flow obstructions. ONSD measurement provides an indirect yet reliable indicator of these ICP variations, as the optic nerve sheath can expand in response to increased pressure. This parameter is particularly useful in differentiating NPH from other forms of hydrocephalus and intracranial hypertension, offering insights into the pathophysiology and aiding in the monitoring of therapeutic interventions. The non-invasive nature and ease of ONSD measurement further enhance its utility in both clinical and research settings, making it a significant adjunct in the comprehensive evaluation of patients with NPH.The exclusion criterion for our feasibility study was the type of implanted valve, specifically requiring a valve with an integrated reservoir chamber. These valves allow assessment of the filling phase. In all patients, the reservoirs refilled rapidly after releasing the reservoir chamber. To utilize this feature as an indicator of a functioning shunt system, we manually compressed the ventricular catheter while pumping the reservoir. This maneuver resulted in sustained emptying of the pump chamber in three-quarters of patients, with rapid refilling after releasing the catheter. However, in about one-quarter of patients, this method was not feasible, leading to immediate reservoir refilling. In cases of slit ventricles or overdrainage, slow reservoir reinflation can be observed. [11, 38] The failure rates of implanted VPS were reported to occur as often as 50% within the first year after implantation and up to 98% within the next 10 years, respectively [3, 7, 12]. The possibility of tumultuous clinical deterioration alongside the sundry symptoms [10–12] related to a shunt malfunction oftentimes result in a hasty vain surgical shunt exploration with a consecutively increased risk of further shunt failure ^2 39^. On average, these circumstances lead to 2.66 revisions per patients, with subsequently increased fears of patients having to undergo another surgical procedure and also a higher risk for subsequent revisions after initial revisions [2, 3, 12, 39, 40]. A key problem in the evaluation of ventricle size is the changed compliance of the ventricular system in long at least since several years implanted VPS patients, leading to only minor changes of the width of the ventricular system [16]. Rocque et al. describe a 93% positive predictive value using shunt tapping to detect a proximal shunt obstruction, independent of implanted systems [41]. However, a consecutive shunt infection remains feared, if performed incorrectly [42].In our pilot feasibility study, we were able to present several new indirect ultrasound imaging signs reflecting an intact and unobstructed VP-shunt system in healthy shunt patients. Pump activation of the valve led to a new occurrence of a free intraabdominal fluid as well as to a consecutive decrease of the ONSD. Furthermore, a flow within the proximal and distal catheter was measured and was used as a surrogate parameter. Further studies are required to evaluate potential changes of flow patterns in dysfunctional shunt systems. Ultimately, the way the pump chamber refills, also after squeezing and releasing the proximal catheter in combination with SMI ultrasound appears to be a promising, non-invasive, yet reliable approach to get relevant information regarding the integrity of the VPS system.

## Strengths and limitations

### Strengths

1. **Innovative Use of SMI Technology**: The application of superb microvascular ultrasound (SMI) for assessing cerebrospinal fluid (CSF) flow in ventriculoperitoneal shunt (VPS) systems represents a novel, non-invasive diagnostic approach. This technique has the potential to enhance the evaluation of shunt function without the need for invasive procedures.
2. **Comprehensive Data Collection**: The study included a detailed analysis of both proximal and distal catheter flow, optic nerve sheath diameter (ONSD), and the presence of intraabdominal fluid, providing a holistic assessment of shunt function.
3. **Clinical Relevance**: By focusing on asymptomatic hydrocephalus patients with VPS, the study addresses a critical need for reliable, non-invasive methods to monitor shunt functionality, potentially reducing the need for exploratory surgeries.

### Limitations

1. **Sample Size**: The number of patients included in this feasibility study is relatively small, which may limit the generalizability of the findings. Larger studies are needed to validate the results and further refine the methodology.
2. **Ultrasound Equipment Quality**: The reliability of our findings is influenced by the quality and capabilities of the ultrasound equipment used. While the Canon Aplio i800 device with an 11 MHz linear array transducer provided valuable data, variations in equipment quality could affect the reproducibility of the results.
3. **Operator Expertise**: The accuracy of SMI and other ultrasound measurements can be highly operator-dependent. The experience and skill of the examining physician play a crucial role in obtaining reliable results. In our study, all examinations were performed by experienced clinicians, but this factor may introduce variability in different clinical settings.
4. **Technical Challenges**: Certain technical aspects, such as the detection of flow direction and the precise measurement of ONSD, posed challenges. The variability in these measurements indicates a need for further standardization and refinement of the examination protocols.Of note, both comparatives analysis of ONSD and other parameters before and after valve activation were unblinded.

## Conclusion

Ultrasound, especially a contemporary technology of superb microvascular ultrasound (SMI) might be a promising and helpful tool to assess the CSF flow within VPS systems and therefore affirm its function in a completely non-invasive manner. Furthermore, appearance of free intraperitoneal fluid followed by repeated compression of a shut reservoir indicates an intact functioning shunt system.

## Data Availability

No datasets were generated or analysed during the current study.

## Abbreviations

CSF: Cerebro Spinal Fluid
CT: Computed Tomography
ICP: Intra Cranial Pressure
MRI: Cranial Magnetic Resonance Imaging
ONSD: Optic Nerve Sheet Diameter
PD: Papilla Diameter
PT: Peri Tonealfluid
SMI: Microvascularultra Sound
SAH: Sub Arachnoid Haemorrhage
US: Ultra Sound
VPS: Ventriculo Peritoneal Shunt
